# ASSOCIATIONS BETWEEN POSTURAL SWAY AND GAIT DOMAINS DURING WALKING WHILE TALKING

**DOI:** 10.1093/geroni/igac059.2600

**Published:** 2022-12-20

**Authors:** Oshadi Jayakody, Helena Blumen, Emmeline Ayers, Cuiling Wang, Joe Verghese

**Affiliations:** Albert Einstein College of Medicine, Bronx, New York, United States; Albert Einstein College of Medicine, Bronx, New York, United States; Albert Einstein College of Medicine, Bronx, New York, United States; Albert Einstein College of Medicine, Bronx, New York, United States; Albert Einstein College of Medicine, Bronx, New York, United States

## Abstract

Poor gait performance during walking-while-talking (WWT) is associated with an increased falls risk. Whether this association between WWT and falls is moderated by balance is not known. To address this, we examined 1) cross-sectional associations between postural sway and gait domains during WWT and, 2) whether postural sway moderated the effects of gait domains on falls. Gait (using a computerized walkway) and postural sway in anteroposterior (AP) and mediolateral (ML) directions (using the SwayStar™ system) during WWT were assessed in 357 community-dwelling older adults (M Age 77.9 years; 55.5% female). Principal component analysis was used to derive distinct gait domains: pace (speed, step length), rhythm (swing, stance, step time) and variability (step length variability, double support time variability). Falls incidence over 12 months were ascertained using bi-monthly calls. Multiple linear regression (adjusted for age, sex, and education) was used to examine the associations between postural sway and gait domains. Poisson regression was used to examine the associations of number of falls with gait domains and postural sway. Greater AP- and ML-sway were associated with slower pace, greater rhythm, and greater variability (p< 0.005). Slower pace (not rhythm or variability) was associated with falls incidence (n=95) and the effect of pace on falls (although p>0.005) was reduced by the ML-sway (β for interaction between pace and sway -0.001 95%CI -0.002, 0.0001). These findings suggest that greater sway during WWT is associated with poorer gait performance. Interventions to maintain ML-sway during WWT may assist with reducing the effects of pace on falls risk.

